# Supplementary material for: Empirical methods for controlling false positives and estimating confidence in ChIP-Seq peaks
Source: BMC Bioinformatics. 2008 Dec 5;9:523. doi: 10.1186/1471-2105-9-523 (PMC2628906; doi:10.1186/1471-2105-9-523)
Supplement: Additional File 1 — A variety of html documents from the USeq web site detailing the available applications, their best usage, output file type descriptions, command line menus, etc. [file 1471-2105-9-523-S1.zip › USeqUserGuides/JavaDoc.zip!/JavaDoc/edu/utah/seq/data/PointData.html]

PointData


|  |  |  |
| --- | --- | --- |
| **Overview** \| **Package** \| **Class** \| **Use** \| **Tree** \| **Deprecated** \| **Index** \| **Help** | |  |
| **PREV CLASS**   **NEXT CLASS** | **FRAMES**    **NO FRAMES**     **All Classes** |  |
| SUMMARY: NESTED \| FIELD \| CONSTR \| METHOD | DETAIL: FIELD \| CONSTR \| METHOD |  |


---


## edu.utah.seq.data Class PointData

```
java.lang.Object
  edu.utah.seq.data.PointData
```

---

``` public class PointData extends java.lang.Object ```

Container for holding genomic positions - scores data.
This data is split by versionedGenome, chromosome, and in some cases strand.
Methods should be created that return a HashMap (ie chr1\_+\_ , PointData)
from various data sources.
Works in tandem with the BarParser to load data on demand.
The number of positions and scores are the same with direct index correspondence.
Positions can be repeated.
Final positions are in interbase coordinates.

---

**Constructor Summary**

`PointData()`

`PointData(java.io.File barFile,
boolean loadPositionScores)`
  
          Reads in a barFile creating a PointData object.


| **Method Summary** | |
| --- | --- |
| `static PointData` | `combinePointData(java.util.ArrayList<PointData> pdAL, boolean nullOriginalPosVal)`             Combines without summing the scores, thus duplicate positions are likely. |
| `static java.util.HashMap<java.lang.String,PointData>[]` | `divide(java.util.HashMap<java.lang.String,PointData> pd, int splitIn)`             Randomly divides data. |
| `void` | `drop(int numToDrop)`             Randomly removes the numToDrop from the position and score arrays. |
| `java.util.HashMap<java.lang.String,java.util.ArrayList<PointData>>` | `fetchPointData(java.io.File directory)`             Loads a directory of xxx.bar PointData files into a HashMap where the key = |
| `static java.util.HashMap<java.lang.String,PointData>[]` | `fetchPointData(java.io.File[] directories, java.io.File[] mergeDirectories, boolean sumScores)`             Multi directory version of fetchPointData(), set mergeDirectories to null to keep everything loaded in memory. |
| `static java.util.HashMap<java.lang.String,PointData>` | `fetchPointData(java.io.File directory, java.io.File mergeDirectory, boolean sumScores)`             Fetches PointData from a directory. |
| `static java.util.HashMap<java.lang.String,PointData>` | `fetchRandomObservations(java.util.HashMap<java.lang.String,PointData> pd, int numWanted)`             Lots of work to fetch random observation. |
| `static java.util.HashMap<java.lang.String,PointData[]>` | `fetchStrandedPointData(java.io.File directory)`             Fetches, but doesn't load or merge, PointData, returning a chromosome split hashmap. |
| `static java.util.HashMap<java.lang.String,PointData[]>[]` | `fetchStrandedPointData(java.io.File[] directories)`             Multi directory version of fetchStrandedPointData(). |
| `int` | `filter(boolean[] maskedBases, double maximumFractionOverlap)`             Takes a boolean[] of masked/true positions and for every position, calculates the overlap of the read. |
| `int[]` | `findIndexes(int startBp, int stopBp)`             Given a start bp (included) and stop bp (not included), returns start (included) and stop (not included) indexes. |
| `static int[]` | `findMinMaxPosition(PointData[] pd)`             Finds the min and maximum positions from all of the position arrays. |
| `BarParser` | `getBarParser()` |
| `Info` | `getInfo()` |
| `int` | `getNumberObservations()` |
| `int[]` | `getPositions()`             Will load positions and scores from file if null. |
| `float[]` | `getScores()`             Will load scores and positions from file if null. |
| `boolean` | `loadPositionScores()`             Gets the BarParser to (re)load the position and scores. |
| `static PointData` | `mergePointData(java.util.ArrayList<PointData> pdAL, boolean nullOriginalPosVal)`             Merges PointData, nulls originals positions and scores, sets strand to '.' if mixed strand. |
| `static java.util.HashMap<java.lang.String,PointData>` | `mergePointData(java.util.HashMap<java.lang.String,PointData>[] hashes, boolean sumScores, boolean nullOriginalPosVal)`             Merges a HashMap by either summing same position scores or simply concatinating then sorting the arrays. |
| `void` | `nullPositionScoreArrays()`             Nulls the position and score arrays to save memory. |
| `boolean` | `pick(int numToPick)`             Randomly picks the numToPick from the position and score arrays and replaces. |
| `void` | `setInfo(Info info)` |
| `void` | `setPositions(int[] position)` |
| `void` | `setScores(float[] score)` |
| `PointData[]` | `split(boolean[] b)`             Splits this.PointData's positions and scores based on the boolean[]. |
| `static java.util.HashMap<java.lang.String,PointData>[]` | `split(java.util.HashMap<java.lang.String,PointData> pd)`             Splits the data in half using random sampling. |
| `static boolean` | `subSample(java.util.HashMap<java.lang.String,PointData> pd, int numPos)`             Reduces the number of observations to the specified number by random selection. |
| `static java.util.HashMap<java.lang.String,PointData>` | `subSamplePercent(java.util.HashMap<java.lang.String,PointData>[] pd, int numPos)`             Will draw the numPos wanted from the array of PointData sampling randomly within but equally throughout. |
| `float` | `sumPositionBP(int startBp, int stopBp)`             Given a start bp (included) and stop bp (not included), returns the sum of the associate positions, the hit count. |
| `float` | `sumScoreBP(int startBp, int stopBp)`             Given a start bp (included) and stop bp (not included), returns the sum of the associate scores. |
| `float` | `sumScoreIndex(int startIndex, int endIndex)`             Given a start index (included) and stop index (NOT included), returns the sum of the associate scores. |
| `static int` | `totalObservations(java.util.HashMap<java.lang.String,PointData> pd)`             Counts the total number of observations. |
| `static int` | `totalObservations(java.util.HashMap<java.lang.String,PointData>[] pd)`             Counts the total number of observations. |
| `static int` | `totalObservations2(java.util.HashMap<java.lang.String,PointData[]> pd)`             Counts the total number of observations. |
| `Point[]` | `windowSum(int startBp, int stopBp, int halfWindowSize)` |
| `boolean` | `writePointData(java.io.File saveDirectory)`             Writes a zip compressed 'chromosome\_strand\_.bar.zip' file into the saveDirectory. |
| `static boolean` | `writePointData(java.util.HashMap<java.lang.String,PointData> pd, java.io.File saveDirectory)`             Writes zip compressed 'chromosome\_strand\_.bar.zip' files into the saveDirectory. |
| `boolean` | `writePointDataToFile(java.io.File barFile)`             Writes a zip compressed 'chromosome\_strand\_.bar.zip' file. |

**Methods inherited from class java.lang.Object**

`equals, getClass, hashCode, notify, notifyAll, toString, wait, wait, wait`

**Constructor Detail**

### PointData

```
public PointData()
```

---


### PointData

```
public PointData(java.io.File barFile,
                 boolean loadPositionScores)
```

:   Reads in a barFile creating a PointData object.

    **Parameters:**: `loadPositionScores` - - set to true to actually load the int[] and float[]s at the time of instantiation. Otherwise they will load with the first call for either of the arrays. Call nullPositionScoreArrays() and System.gc() when done to save memory.


**Method Detail**

### writePointData

```
public boolean writePointData(java.io.File saveDirectory)
```

:   Writes a zip compressed 'chromosome\_strand\_.bar.zip' file into the saveDirectory.
    ie. chr21\_+\_.bar.zip
    If strand is "." writes just chr21.bar.zip
    Will over write an existing file in the saveDirectory.

---


### writePointDataToFile

```
public boolean writePointDataToFile(java.io.File barFile)
```

:   Writes a zip compressed 'chromosome\_strand\_.bar.zip' file.
    Will over write an existing file in the saveDirectory.

---


### writePointData

```
public static boolean writePointData(java.util.HashMap<java.lang.String,PointData> pd,
                                     java.io.File saveDirectory)
```

:   Writes zip compressed 'chromosome\_strand\_.bar.zip' files into the saveDirectory.
    ie. chr21\_+\_.bar.zip
    If strand is "." writes just chr21.bar.zip
    Will over write existing files!

---


### filter

```
public int filter(boolean[] maskedBases,
                  double maximumFractionOverlap)
```

:   Takes a boolean[] of masked/true positions and for every position, calculates the overlap of the read.
    If the overlap exceeds the max then it is tossed. Leaves masked data in memory.

---


### loadPositionScores

```
public boolean loadPositionScores()
```

:   Gets the BarParser to (re)load the position and scores.

---


### nullPositionScoreArrays

```
public void nullPositionScoreArrays()
```

:   Nulls the position and score arrays to save memory.
    These will reload from file if you call for them after nulling.

---


### findMinMaxPosition

```
public static int[] findMinMaxPosition(PointData[] pd)
```

:   Finds the min and maximum positions from all of the position arrays.
    Will load the data if not already loaded.

    :   **Returns:**: int[2] {min, max}

---


### sumScoreBP

```
public float sumScoreBP(int startBp,
                        int stopBp)
```

:   Given a start bp (included) and stop bp (not included), returns the sum of the associate scores.

---


### sumPositionBP

```
public float sumPositionBP(int startBp,
                           int stopBp)
```

:   Given a start bp (included) and stop bp (not included), returns the sum of the associate positions, the hit count.

---


### findIndexes

```
public int[] findIndexes(int startBp,
                         int stopBp)
```

:   Given a start bp (included) and stop bp (not included), returns start (included) and stop (not included) indexes.

---


### sumScoreIndex

```
public float sumScoreIndex(int startIndex,
                           int endIndex)
```

:   Given a start index (included) and stop index (NOT included), returns the sum of the associate scores.

---


### windowSum

```
public Point[] windowSum(int startBp,
                         int stopBp,
                         int halfWindowSize)
```

---


### divide

```
public static java.util.HashMap<java.lang.String,PointData>[] divide(java.util.HashMap<java.lang.String,PointData> pd,
                                                                     int splitIn)
```

:   Randomly divides data.

    :   **Parameters:**: `splitIn` - should be 2 or 1/2, 3 for 1/3rds etc.

---


### split

```
public static java.util.HashMap<java.lang.String,PointData>[] split(java.util.HashMap<java.lang.String,PointData> pd)
```

:   Splits the data in half using random sampling.
    Will first call PointData.subSample() if not evenly divisible by 2.

---


### split

```
public PointData[] split(boolean[] b)
```

:   Splits this.PointData's positions and scores based on the boolean[].

    :   **Parameters:**: `b` - -must be same length as this.positions or returns null **Returns:**: two new PointDatas with info of parent, watch it, these reference the parent's info.

---


### fetchRandomObservations

```
public static java.util.HashMap<java.lang.String,PointData> fetchRandomObservations(java.util.HashMap<java.lang.String,PointData> pd,
                                                                                    int numWanted)
```

:   Lots of work to fetch random observation.
    Returns null if you ask for more observations that present.

---


### subSample

```
public static boolean subSample(java.util.HashMap<java.lang.String,PointData> pd,
                                int numPos)
```

:   Reduces the number of observations to the specified number by random selection. This does not work with
    drastic sub samplings, ie > 1/2 the data. Becomes inefficient, use

---


### subSamplePercent

```
public static java.util.HashMap<java.lang.String,PointData> subSamplePercent(java.util.HashMap<java.lang.String,PointData>[] pd,
                                                                             int numPos)
```

:   Will draw the numPos wanted from the array of PointData sampling randomly within but equally throughout.

---


### drop

```
public void drop(int numToDrop)
```

:   Randomly removes the numToDrop from the position and score arrays.
    Use to shrink the size of the data.

---


### pick

```
public boolean pick(int numToPick)
```

:   Randomly picks the numToPick from the position and score arrays and replaces.

---


### totalObservations

```
public static int totalObservations(java.util.HashMap<java.lang.String,PointData> pd)
```

:   Counts the total number of observations.
    This does not load the data.

---


### totalObservations2

```
public static int totalObservations2(java.util.HashMap<java.lang.String,PointData[]> pd)
```

:   Counts the total number of observations.
    This does not load the data.

---


### totalObservations

```
public static int totalObservations(java.util.HashMap<java.lang.String,PointData>[] pd)
```

:   Counts the total number of observations.
    This does not load the data.

---


### combinePointData

```
public static PointData combinePointData(java.util.ArrayList<PointData> pdAL,
                                         boolean nullOriginalPosVal)
```

:   Combines without summing the scores, thus duplicate positions are likely.

---


### mergePointData

```
public static java.util.HashMap<java.lang.String,PointData> mergePointData(java.util.HashMap<java.lang.String,PointData>[] hashes,
                                                                           boolean sumScores,
                                                                           boolean nullOriginalPosVal)
```

:   Merges a HashMap by either summing same position scores or simply concatinating then sorting the arrays.

---


### mergePointData

```
public static PointData mergePointData(java.util.ArrayList<PointData> pdAL,
                                       boolean nullOriginalPosVal)
```

:   Merges PointData, nulls originals positions and scores, sets strand to '.' if mixed strand.

---


### fetchPointData

```
public java.util.HashMap<java.lang.String,java.util.ArrayList<PointData>> fetchPointData(java.io.File directory)
```

:   Loads a directory of xxx.bar PointData files into a HashMap where the
    key =

---


### fetchPointData

```
public static java.util.HashMap<java.lang.String,PointData>[] fetchPointData(java.io.File[] directories,
                                                                             java.io.File[] mergeDirectories,
                                                                             boolean sumScores)
```

:   Multi directory version of fetchPointData(), set mergeDirectories to null to keep everything loaded in memory.

---


### fetchStrandedPointData

```
public static java.util.HashMap<java.lang.String,PointData[]>[] fetchStrandedPointData(java.io.File[] directories)
```

:   Multi directory version of fetchStrandedPointData(). Fetches, but doesn't load or merge, PointData
    Returns null if no PointData files found.

---


### fetchStrandedPointData

```
public static java.util.HashMap<java.lang.String,PointData[]> fetchStrandedPointData(java.io.File directory)
```

:   Fetches, but doesn't load or merge, PointData, returning a chromosome split hashmap.
    Returns null if no PointData files found.

---


### fetchPointData

```
public static java.util.HashMap<java.lang.String,PointData> fetchPointData(java.io.File directory,
                                                                           java.io.File mergeDirectory,
                                                                           boolean sumScores)
```

:   Fetches PointData from a directory. If same chromosome found, ie from a stranded set, it will merge the data.
    If a mergeDirectory is provided, the merged data will be written to the directory otherwise it is kept in memory.

    :   **Parameters:**: `mergeDirectory` - set to null to keep merged data in memory, otherwise it will be written to disk.

---


### getInfo

```
public Info getInfo()
```

---


### setInfo

```
public void setInfo(Info info)
```

---


### getPositions

```
public int[] getPositions()
```

:   Will load positions and scores from file if null.

---


### setPositions

```
public void setPositions(int[] position)
```

---


### getScores

```
public float[] getScores()
```

:   Will load scores and positions from file if null.

---


### setScores

```
public void setScores(float[] score)
```

---


### getNumberObservations

```
public int getNumberObservations()
```

---


### getBarParser

```
public BarParser getBarParser()
```


---


|  |  |  |
| --- | --- | --- |
| **Overview** \| **Package** \| **Class** \| **Use** \| **Tree** \| **Deprecated** \| **Index** \| **Help** | |  |
| **PREV CLASS**   **NEXT CLASS** | **FRAMES**    **NO FRAMES**     **All Classes** |  |
| SUMMARY: NESTED \| FIELD \| CONSTR \| METHOD | DETAIL: FIELD \| CONSTR \| METHOD |  |


---
